# A Post-Quantum Public-Key Signcryption Scheme over Scalar Integers Based on a Modified LWE Structure

**DOI:** 10.3390/s25154728

**Published:** 2025-07-31

**Authors:** Mostefa Kara, Mohammad Hammoudeh, Abdullah Alamri, Sultan Alamri

**Affiliations:** 1 Interdisciplinary Research Center for Intelligent Secure Systems, King Fahd University of Petroleum and Minerals, Dhahran 31261, Saudi Arabia; 2 Information and Computer Science Department, King Fahd University of Petroleum and Minerals, Dhahran 31261, Saudi Arabia; mohammad.hammoudeh@kfupm.edu.sa; 3 College of Computing Science and Engineering, University of Jeddah, Jeddah 23218, Saudi Arabia; amalamri@uj.edu.sa; 4 College of Computing and Informatics, Saudi Electronic University, Riyadh 93499, Saudi Arabia; salamri@seu.edu.sa

**Keywords:** light IoT, LWE, signcryption, post-quantum, scalar integer, cryptosystem

## Abstract

To ensure confidentiality and integrity in the era of quantum computing, most post-quantum cryptographic schemes are designed to achieve either encryption or digital signature functionalities separately. Although a few signcryption schemes exist that combine these operations into a single, more efficient process, they typically rely on complex vector, matrix, or polynomial-based structures. In this work, a novel post-quantum public-key encryption and signature (PQES) scheme based entirely on scalar integer operations is presented. The proposed scheme employs a simplified structure where the ciphertext, keys, and core cryptographic operations are defined over scalar integers modulo *n*, significantly reducing computational and memory overhead. By avoiding high-dimensional lattices or ring-based constructions, the PQES approach enhances implementability on constrained devices while maintaining strong security properties. The design is inspired by modified learning-with-errors (LWE) assumptions, adapted to scalar settings, making it suitable for post-quantum applications. Security and performance evaluations, achieving a signcryption time of 0.0007 s and an unsigncryption time of 0.0011 s, demonstrate that the scheme achieves a practical balance between efficiency and resistance to quantum attacks.

## 1. Introduction

The rapid advancement of quantum computing presents a profound challenge to classical cryptographic systems. Algorithms such as Shor’s and Grover’s are expected to efficiently solve hard mathematical problems that underpin the security of widely used schemes, such as RSA, ECC, and DSA [1,2]. Specifically, quantum algorithms can break encryption systems based on integer factorization and discrete logarithms in polynomial time, rendering many conventional public-key infrastructures vulnerable [3,4,5].

In response to this growing threat, the field of post-quantum cryptography (PQC) has emerged, focusing on designing cryptographic primitives that remain secure in the presence of quantum adversaries. Most PQC efforts have concentrated on either encryption or signature schemes [6], with only a limited number of integrated signcryption schemes proposed. These existing schemes often rely on high-dimensional structures such as vectors, matrices, or ring polynomials, which can introduce complexity and overhead in practical implementations.

Such high-dimensional algebraic structures, commonly used in lattice-based schemes, typically involve intensive linear algebra operations. These operations are computationally expensive and require significant memory and storage resources, making them less ideal for constrained environments such as embedded systems or IoT devices. To address this issue, scalar integer arithmetic has been adopted, which significantly reduces algorithmic complexity by avoiding the overhead of large matrix operations. Scalar-based computations are easier to implement, require fewer resources, and are better suited for low-power hardware while still enabling strong security through properly structured hardness assumptions.

This paper proposes a novel post-quantum public-key encryption and signature (PQES) scheme that unifies both functionalities within a single, scalar-based cryptographic structure. Unlike traditional LWE-based systems, which typically involve large matrix operations [7], this construction utilizes a modified version of the learning-with-errors (LWE) problem, adapted to scalar integer arithmetic modulo *n*. This scalar-only design simplifies computation, reduces memory requirements, and enhances feasibility for resource-constrained environments, without compromising on security.

The proposed scheme contributes to the post-quantum landscape in three key ways. First, it removes the need for high-dimensional algebraic structures, resulting in a more accessible implementation for lightweight or embedded systems. Second, by leveraging a scalar-based adaptation of LWE, the scheme retains strong theoretical foundations while opening new directions for PQC research beyond the conventional matrix and lattice-based settings. Third, the dual functionality of encryption and signing in a single operation promotes efficiency and security in scenarios where both confidentiality and authenticity are critical. Taken together, these advantages make the PQES scheme a compelling candidate for future quantum-resilient digital infrastructures.

## 2. Related Work

Post-quantum cryptography (PQC) is currently at the forefront of cryptographic research, driven by the impending threat posed by quantum computers to classical cryptographic schemes [8]. Shor’s and Grover’s algorithms render numerous traditional systems, including RSA, Diffie–Hellman, and ECC, vulnerable by reducing the complexity of their underlying hard problems to polynomial time on quantum hardware. Consequently, the National Institute of Standards and Technology (NIST) has initiated and progressed through several rounds of standardization for quantum-resistant algorithms. This effort has led to a rich taxonomy of post-quantum schemes, including lattice-based, code-based, hash-based, multivariate, and isogeny-based cryptosystems [9].

This section provides an extensive analysis of prominent cryptographic schemes categorized by their security goals (encryption, digital signature, or signcryption), underlying mathematical structure, and quantum resistance. In particular, we focus on the design decisions, strengths, and limitations of selected state-of-the-art solutions and closely examine integrated schemes such as signcryption that aim to provide both confidentiality and authenticity within a single framework.

### 2.1. Lattice-Based Cryptographic Schemes

Lattice-based cryptography is widely regarded as one of the most promising approaches for post-quantum security due to its well-studied hardness assumptions, such as the shortest vector problem (SVP) and learning with errors (LWE). Among the lattice-based schemes standardized or shortlisted by the NIST are CRYSTALS-Kyber for encryption and CRYSTALS-Dilithium and Falcon for digital signatures.

CRYSTALS-Kyber [10] is a key encapsulation mechanism (KEM) based on Module-LWE. It has become the most prominent choice for post-quantum encryption because of its strong security guarantees and practical efficiency. Kyber employs a compact and structured lattice framework that allows for fast key generation, encryption, and decryption operations. Its parameters are tuned to achieve IND-CCA2 security in the standard model, and its modular nature facilitates implementation across a wide range of platforms. In terms of efficiency, Kyber outperforms other lattice-based KEMs and shows excellent performance metrics on both general-purpose CPUs and constrained microcontrollers. This performance, coupled with its provable security based on hard lattice problems, makes Kyber a suitable candidate for large-scale deployment in post-quantum infrastructures.

CRYSTALS-Dilithium and Falcon: CRYSTALS-Dilithium [11], closely related to Kyber, is a lattice-based digital signature scheme built upon Module-LWE and Module-SIS problems. Its design focuses on side-channel resistance and practical efficiency, making it a compelling choice for quantum-resistant digital signatures. Falcon [12], on the other hand, adopts a more mathematically intricate structure known as the NTRU lattice. It uses fast Fourier transforms (FFTs) for signature generation and verification, resulting in highly compact signatures. Falcon’s security is provable in the tightest sense under lattice assumptions, and it remains one of the few schemes that offers both high performance and a small footprint in terms of key and signature sizes. Although both Dilithium and Falcon are quantum-resistant, their operational characteristics differ: Dilithium offers more stable performance and simpler implementations, while Falcon provides tighter security reductions at the cost of more complex arithmetic.

### 2.2. Code-Based Cryptographic Schemes

Code-based cryptography is among the oldest post-quantum paradigms, with McEliece encryption first proposed in the 1970s. The underlying assumption is the hardness of decoding a random linear code, a problem that has withstood decades of cryptanalytic scrutiny, even in the presence of quantum adversaries.

Classic McEliece [13] is a code-based public-key encryption scheme selected by the NIST for standardization. It relies on the difficulty of decoding general linear codes, particularly Goppa codes. McEliece offers exceptionally fast encryption and decryption operations, especially in high-throughput environments. However, its primary drawback is the unusually large public key size of several hundred kilobytes, which may be a limiting factor for embedded and bandwidth-constrained devices. Despite this limitation, McEliece remains one of the most conservative and secure choices for quantum-resistant encryption, benefiting from over four decades of cryptanalytic resilience.

BIKE and HQC: BIKE (bit flipping key encapsulation) and HQC (hamming quasi-cyclic) are code-based KEMs that have been shortlisted in NIST’s PQC process [14]. Both schemes operate on quasi-cyclic codes, which reduce the key size compared to McEliece and improve practicality. BIKE leverages a bit-flipping decoder that offers lightweight computations, making it suitable for constrained environments. HQC, meanwhile, emphasizes strong theoretical underpinnings and compact key sizes while retaining code-based hardness assumptions. Both are designed to resist quantum attacks and offer IND-CCA2 security in the ROM (random oracle model).

### 2.3. Hash-Based Cryptographic Schemes

Hash-based signature schemes are another class of PQC primitives, offering security based on the collision resistance of cryptographic hash functions, a well-understood and widely deployed class of primitives.

SPHINCS+ is a stateless hash-based digital signature scheme, recognized as a strong post-quantum candidate due to its minimal reliance on algebraic structures [15]. It avoids the pitfalls of structured lattices and algebraic codes by relying entirely on secure hash functions and Merkle tree constructions. Although SPHINCS+ tends to have relatively large signature sizes (tens of kilobytes), its conservative design and long-standing theoretical security make it a robust choice for applications where signature size is not a critical concern. Its statelessness also makes implementation less error-prone, a valuable feature in real-world deployment.

### 2.4. Isogeny-Based Cryptography

Isogeny-based cryptography is a relatively novel area in PQC that offers some of the smallest key and ciphertext sizes among all post-quantum approaches. However, its computational complexity is generally higher, and its mathematical foundations are still being actively researched.

Dey et al. [16] proposed a signcryption scheme using supersingular isogeny-based constructions. Their design relies on three novel hard problems: the commutative supersingular isogeny decisional Diffie–Hellman (CSIDH-DDH), the group action inverse problem (GAIP), and the commutative supersingular isogeny knowledge of exponent (CSIKoE). This scheme achieves quantum resistance by avoiding known polynomial-time quantum algorithms for these problems. Additionally, isogeny-based systems tend to provide relatively compact keys and ciphertexts.

### 2.5. Scalar Integer Approaches

Signcryption is a cryptographic primitive that aims to simultaneously provide message confidentiality and authenticity in a single logical step. While this concept reduces the overhead of separately executing encryption and signature algorithms, it has not yet been widely explored in the post-quantum domain. Several classical signcryption schemes rely on scalar integer arithmetic, elliptic curves, or other conventional assumptions that may not be quantum-safe.

Kara et al. [17] introduced a symmetric cryptosystem that fuses encryption and signature capabilities using scalar integer operations. Their approach modifies traditional symmetric encryption to embed signature data implicitly within the ciphertext structure. Linear construction leverages variables from both encryption and signing mechanisms, ensuring minimal overhead while satisfying authenticity and confidentiality requirements. This design represents a lightweight solution suitable for constrained devices but lacks post-quantum resistance due to its reliance on discrete logarithm-based scalar arithmetic. Moreover, as a symmetric scheme, its practical applicability in open, public-key settings remains limited.

Kasyoka et al. [18] provided a critical cryptanalysis of a certificateless signcryption scheme originally proposed by Wei and Ma. While the original scheme claims security under the elliptic curve computational Diffie–Hellman (EC-CDH) assumption and avoids pairings, the authors demonstrated its vulnerability to public key replacement attacks, a significant concern in certificateless settings. The proposed improvement attempts to harden the scheme against such attacks. However, both the original and revised constructions remain rooted in scalar elliptic curve arithmetic, rendering them susceptible to quantum attacks due to Shor’s algorithm. Thus, despite their efficient construction and certificateless benefits, these schemes are not quantum-resistant and are unlikely to be viable in the long term.

From Table 1 and the previous analysis, several trends and gaps can be identified, including the dominance of lattice-based designs. Lattice-based schemes dominate the PQC landscape, especially for encryption and signatures. Their provable security under worst-case lattice assumptions and their operational efficiency make them leading candidates in standardization efforts. While encryption and digital signature schemes are being standardized, signcryption, despite its theoretical and practical appeal, remains underrepresented. Among the listed schemes, only Dey et al.’s isogeny-based proposal explicitly targets quantum resistance in a signcryption setting. Inefficiency of classical signcryption approaches, including many classical signcryption schemes that rely on scalar integer or elliptic curve arithmetic (e.g., Refs. [17,18]), offer practical advantages but do not provide adequate quantum resistance, making them unsuitable for future-proof systems. Although still under development, isogeny-based cryptography holds promise due to its small key sizes and emerging quantum resistance. However, its computational costs and recent cryptanalytic advances highlight the need for caution and further research.

In light of the gaps identified above, the proposed PQES (post-quantum encryption and signature) scheme introduces a novel scalar-integer-based signcryption design that is lightweight and quantum-resistant. While existing scalar-based schemes are either non-quantum-safe or purely symmetric, this work employs modified LWE constructions adapted to operate over scalar integers modulo *n*. It achieves the dual goals of efficiency and post-quantum security. Furthermore, its design sidesteps the heavy algebraic machinery of high-dimensional lattices, rings, and isogenies, making it more suitable for constrained environments. PQES serves as a potential stepping stone toward standardizing practical signcryption systems for the post-quantum era.

## 3. Conventional LWE Scheme

In the setup and key generation stage, the user randomly picks *a* a vector, *e* a vector of small errors, and *s* the secret key vector. The result is the public key (a,b).

(1) (a,b=a·s+e)

In the encryption stage of a *t*-bit binary plain text, classical LWE utilizes three randomly generated small variables, $r,e_{1},e_{2}$, for each bit.

$$\left\{\begin{array}{c} u=a\cdot r+e_{1}\\ v=b\cdot r+e_{2}+z\times \left\lfloor p/2\right\rfloor \end{array}\right.$$

where “z=0” or “z=1” is the encrypted message.

In the decryption stage, the receiver calculates $m^{\prime }$ and then evaluates it.

(2) $$m^{\prime }=v-u\cdot s$$

$$z=\left\{\begin{array}{c} 0\;\mathrm{if}\;m^{\prime }\;\mathrm{is}\;\mathrm{closer}\;\mathrm{to}\;0\\ 1\;\mathrm{if}\;m^{\prime }\;\mathrm{is}\;\mathrm{closer}\;\mathrm{to}\;\left\lfloor p/2\right\rfloor \end{array}\right.$$

In contrast to traditional LWE-based methods that rely on vectors and matrices for secret keys, public keys, and ciphertexts, PQES uses scalar integers, leading to simpler computations and enhanced efficiency. By representing encryption components as scalar values and integrating the signature directly, the scheme lowers computational complexity while maintaining robust security properties. This design results in a more efficient cryptographic system that remains resilient against quantum threats.

## 4. Proposed Signcryption Scheme

### 4.1. Basic Proposal

PQES has, globally, the same key generation process as ford the basic LWE cryptography (1), except it uses large numbers (*a*, *s*, *e*) instead of vectors and matrices.

To encrypt the whole message *m*, two large random numbers $r_{1},r_{2}\in Z$ are first generated, and then the cipher (*u*, *v*) is computed as follows:

Enc(m):

(3) $$\left\{\begin{array}{c} u=r_{1}\left(a-m\right)\;\mathrm{mod}\;p\\ v=r_{1}\left(b+r_{2}\right)\;\mathrm{mod}\;p \end{array}\right.$$

### 4.2. Enhanced Proposal

To sign the encrypted message, an enhanced version of the proposal is introduced. The random variables $r_{1}$ and $r_{2}$ have been played with.

(4) $$\left\{\begin{array}{c} r_{1}=f\left(h,sk,\sigma \right)\\ r_{2}=f\left(h,sk,\sigma ,r_{1}\right) \end{array}\right.$$

where *h* is the plain text’s hash, sk are sender secret keys, and σ is the signature. From (4), $\left(r_{1},r_{2}\right)$ serve as the subsignature due to implicating the message hash and sender secret keys.

Algorithms 1 and 2 present the encryption-signing and decryption-verification operations, respectively.

#### 4.2.1. Key Gen Function

Public parameters: $PP:(p,p^{\prime })$ are two prime numbers.

Receiver secret key: $SK_{r}:\left(s,e\right)$ are two large random numbers.

Receiver public key: $PK_{r}:\left(a,b\right)$ are two random numbers, where *b* is defined in (1) over scalar integers.

Sender secret key: $SK_{s}:\left(k_{1},k_{2}\right)$ are random numbers.

Sender public key: $PK_{s}:\left(K_{11},K_{12}\right)$ is computed as follows ($\;\mathrm{mod}\;p^{\prime }$).

$$\left\{\begin{array}{c} K_{11}=k_{1}^{k_{1}}\;\mathrm{mod}\;p^{\prime },\\ K_{12}=k_{1}^{k_{2}}\;\mathrm{mod}\;p^{\prime }, \end{array}\right.$$

**Algorithm 1** Encryption &Sing: Sender**Require:** $m,PP,PK_{r},SK_{s}$1:**function** Enc&Sig2:      $h\leftarrow Hash\left(m\right)\;\mathrm{mod}\;p^{\prime }$3:      $h^{\prime }\leftarrow Hash\left(h\right)\;\mathrm{mod}\;p^{\prime }$4:      $x↚Z_{p^{\prime }}$5:      $a_{2},b_{2}\leftarrow RFrag\left(k_{2}\right)$       ▹ random fragmentation of $k_{2}$ where $k_{2}=a_{2}+b_{2}$6:      $a_{1}\leftarrow k_{1}-a_{2}\;\mathrm{mod}\;p^{\prime }-1$7:      $b_{1}\leftarrow k_{1}-b_{2}\;\mathrm{mod}\;p^{\prime }-1$8:      $s_{1}\leftarrow k_{1}^{ha_{1}+k_{1}x}\;\mathrm{mod}\;p^{\prime }$9:      $r_{1}\leftarrow k_{1}^{hb_{1}+k_{1}\left(s_{1}+h^{\prime }-x\right)}\;\mathrm{mod}\;p^{\prime }$10:    $u\leftarrow r_{1}\left(a-m\right)\;\mathrm{mod}\;p$11:    $s_{2}\leftarrow uk_{1}^{ha_{2}+k_{1}\left(r_{1}-x\right)}\;\mathrm{mod}\;p^{\prime }$12:    $r_{2}\leftarrow s_{1}^{s_{2}}k_{1}^{hb_{2}+k_{1}\left(s_{1}-h^{\prime }+r_{1}+x\right)}\;\mathrm{mod}\;p^{\prime }$13:    $v\leftarrow r_{1}\left(b+r_{2}\right)\;\mathrm{mod}\;p$14:    c←(u,v)15:    $\sigma \leftarrow (s_{1},s_{2})$16:    return (c,σ)17:**end function**

**Algorithm 2** Decryption&Verf: Receiver**Require:** $c,\sigma ,PP,SK_{r},PK_{s}$1:**function** Dec&Verf2:      d←(v−us)modp3:      $r_{1}\leftarrow d÷e$4:      $d^{\prime }\leftarrow \left(d\;\mathrm{mod}\;e\right)r_{1}^{-}\;\mathrm{mod}\;p$       ▹$r^{-}$ is multiplicative inverse of $r_{1}$ base *p*5:      $m\leftarrow d^{\prime }÷s$6:      $r_{2}\leftarrow d^{\prime }\;\mathrm{mod}\;s$7:      $h\leftarrow Hash\left(m\right)\;\mathrm{mod}\;p^{\prime }$8:      $h^{\prime }\leftarrow Hash\left(h\right)\;\mathrm{mod}\;p^{\prime }$9:      if $\;r_{1}r_{2}\;\mathrm{mod}\;p^{\prime }\neq s_{1}^{s_{2}}K_{11}^{h+2s_{1}+r_{1}}\;\mathrm{mod}\;p^{\prime }\;:\;return\;⊥$10:    if $\;s_{1}s_{2}\;\mathrm{mod}\;p^{\prime }\neq uK_{11}^{r_{1}+h}\;\mathrm{mod}\;p^{\prime }\;:\;return\;⊥$11:    if $\;s_{1}r_{1}K_{12}^{h}\;\mathrm{mod}\;p^{\prime }\neq K_{11}^{s_{1}+h^{\prime }+2h}\;\mathrm{mod}\;p^{\prime }\;:\;return\;⊥$12:    if $\;s_{2}r_{2}\;\mathrm{mod}\;p^{\prime }\neq us_{1}^{s_{2}}K_{11}^{s_{1}+2r_{1}-h^{\prime }}K_{12}^{h}\;\mathrm{mod}\;p^{\prime }\;:\;return\;⊥$13:    return (m,1)14:**end function**

#### 4.2.2. Signcryption and Unsigncryption Operations

Algorithms 1 and 2 define the core operations of the PQES scheme, merging encryption and signature in a unified process using scalar arithmetic.

During encryption and signing (Algorithm 1), the sender computes a hashed version of the message *m* and derives a nested signature $\left(s_{1},s_{2}\right)$ that implicitly involves the sender’s secret key and the message hash. This process uses fragmented parts of the secret key $k_{2}=a_{2}+b_{2}$, which are integrated into the exponentiation steps to ensure non-trivial binding to the specific message and sender identity. The outputs $\left(r_{1},r_{2}\right)$ function as subsignatures, securely attached to the hash and secret parameters. The ciphertext c=(u,v) is also constructed using $r_{1}$ to encode both the message and signature dependencies in scalar form.

In decryption and verification (Algorithm 2), the receiver extracts the values $\left(r_{1},r_{2}\right)$ from the ciphertext using the secret key pair (s,e) and reconstructs the message *m* and intermediate hash values. A series of verification checks ensures the integrity and authenticity of the message by validating algebraic relationships among the signature components $\left(s_{1},s_{2}\right)$, the public keys $\left(K_{11},K_{12}\right)$, and the recovered values. These equations enforce a strong linkability between the encrypted message and the signer’s secret key, protecting against forgery or tampering.

The algorithms are designed to satisfy the core security goals of confidentiality, authenticity, and integrity. Confidentiality is ensured by masking the plain text message *m* within the ciphertext components (u,v), using both the receiver’s public key and randomness derived from $r_{1}$. Authenticity is enforced through tightly coupled signature components ($s_{1},s_{2}$), which are derived using the sender’s secret keys and message hash, making it infeasible for an adversary to forge valid outputs without access to these secrets. Integrity is preserved by multiple verification checks in the decryption phase, which validate algebraic relationships between the signature, public keys, and reconstructed message. Any tampering with the ciphertext or signature components will cause these checks to fail, ensuring that the message received is exactly the one signed and encrypted by the legitimate sender.

#### 4.2.3. Computation and Parameter Selection

To ensure correct operations, the following points must be satisfied.

- $c_{1}$; $p^{\prime }<s$: This is to ensure decryption. After removal of $r_{1}$, (ms+r2) is extracted, so $r_{2}\approx p^{\prime }$ should be less than *s*.
- $c_{2}$; $p^{\prime }ms<e$: This is to ensure decryption. After v−us is computed, $r_{1}e+r_{1}r_{2}+r_{1}sm$ is extracted, where $r_{1}r_{2}<r_{1}sm$. As a result, to calculate $r_{1}$, $r_{1}sm$ should be less than *e*; with $r_{1}\approx p^{\prime }$, $p^{\prime }ms$ should be less than *e*. Suppose that *M* is the maximum size of encrypted messages; the receiver chooses *e* where $p^{\prime }Ms<e$.
- $c_{3}$; $p^{\prime }e<p$: This is to ensure decryption by eliminating modp.
- $c_{4}$; p<a: This is to employ the mod in b=(as+e)modp and hide *s*, *e* perfectly.
- $c_{5}$; $k_{i}\approx p^{\prime };$: This is to ensure the desired security level.
- $c_{6}$; $p^{\prime }$ should be large enough: This is to face a brute force attack and ensure security.

#### 4.2.4. Correctness Proof

**Lemma 1.**  *The decryption would be correct if $c_{1}$, $c_{2}$, and $c_{3}$ hold.*

**Proof.**  In line 2, $d=v-us=r_{1}\left(b+r_{2}\right)-r_{1}\left(a-m\right)s$, sine b=as+e,

$$d=r_{1}as+r_{1}e+r_{1}r_{2}-r_{1}as+r_{1}ms=r_{1}e+r_{1}r_{2}+r_{1}ms,$$

according to $c_{2}$ and $c_{3}$, $d÷e=r_{1}$ where ÷ is an integer division.

$$d^{\prime }=\left(d\;\mathrm{mod}\;e\right)r_{1}^{-}=\left(r_{1}r_{2}+r_{1}ms\right)r_{1}^{-}=r_{2}+ms,$$

according to $c_{1}$, $d^{\prime }\;\mathrm{mod}\;s=r_{2}$ and $d^{\prime }÷s=m$. □

**Lemma 2.**  *Given a valid sender public key $PK_{s}$, a correct ciphertext c=(u,v), a matching signature $\sigma =(s_{1},s_{2})$, and a related signature–subsignature σ−r where r presented by $\left(r_{1},r_{2}\right)$, Algorithm 2 returns (m,1).*

**Proof.**  As for line 9,

$$r_{1}r_{2}=k_{1}^{hb_{1}+k_{1}\left(s_{1}+h^{\prime }-x\right)}s_{1}^{s_{2}}k_{1}^{hb_{2}+k_{1}\left(s_{1}-h^{\prime }+r_{1}+x\right)}$$

$$=s_{1}^{s_{2}}k_{1}^{h\left(b_{1}+b_{2}\right)+k_{1}\left(2s_{1}+r_{1}\right)},$$

with $b_{1}+b_{2}=k_{1}$ and $k_{1}^{k_{1}}=K_{11}$, $r_{1}r_{2}=s_{1}^{s_{2}}K_{11}^{h+2s_{1}+r_{1}}$.

As for line 10,

$$s_{1}s_{2}=k_{1}^{ha_{1}+k_{1}x}uk_{1}^{ha_{2}+k_{1}\left(r_{1}-x\right)}$$

$$=uk_{1}^{h\left(a_{1}+a_{2}\right)+k_{1}r_{1}},$$

with $a_{1}+a_{2}=k_{1}$, $s_{1}s_{2}=uK_{11}^{r_{1}+h}$

The same applies for lines 11 and 12. □

## 5. Security Analysis

### 5.1. One-Way with Ambiguity (OWA) Assumption

Let *p* be a large prime. By defining the function

f(x;y,z)=(xymodp,xzmodp)

we suppose an oracle samples random $x,y,z\in Z_{p}^{*}$ and outputs (a,b)=(xymodp,xzmodp). Then,

Given $\left(a,b\right)\in Z_{p}^{*}\times Z_{p}^{*}$, it is computationally infeasible to distinguish the original hidden preimage (x,y,z) from the set of all (p−1) valid preimages without additional information.

This assumption does not assert that computing a valid solution is hard (as all can be computed efficiently) but rather that distinguishing the correct one is infeasible.

The OWA assumption provides a basis for designing cryptographic primitives where many valid preimages exist for the same output; only one preimage is correct in context (e.g., for key derivation or authentication), and the adversary cannot identify the correct preimage without extra distinguishing information.

Such systems benefit from preimage ambiguity, which can enhance privacy, obfuscation, or identity-hiding properties.

### 5.2. Secure Ciphertext

The ciphertext c=(u,v) can be represented as follows:

$$\left\{\begin{array}{c} xy\;\mathrm{mod}\;p=a\\ xz\;\mathrm{mod}\;p=b \end{array}\right.$$

where *p* is a large prime, $a,b\in Z_{p}^{*}$ are known, and $x,y,z\in Z_{p}^{*}$ are unknown.

This system has exactly p−1 valid solutions for $x\in Z_{p}^{*}$, each associated with a unique pair (y,z) such that

$$y=ax^{-1}\;\mathrm{mod}\;p,\;z=bx^{-1}\;\mathrm{mod}\;p$$

Based on the OWA assumption, the attacker can compute all p−1 possible valid tuples (x,y,z). However, without additional information, distinguishing the original or correct tuple used in the protocol is computationally infeasible.

### 5.3. Secure Receiver’s Public Key

The secret keys *e* and *s* are safe due to the existence of many pairs, especially with large enough numbers. $\left(e^{\prime },s^{\prime }\right)$ satisfies the equation $b=as^{\prime }+e^{\prime }\;\mathrm{mod}\;p$, where the attacker has only the public key (a,b) and knows *p*. Therefore, pk is of the following form:

ax+bymodp=c

Under the same assumption above (OWA), this equation has p−1 possible solutions, where there is only one (s,e) that is correct, but there is no effective way to determine it.

### 5.4. Secure Sender’s Public Key

The sender’s public key is expressed using two exponentiations of the form $x^{x}$ and $x^{y}$, where *x* and *y* are large and randomly chosen secret values. This construction ensures that both the base and the exponent in each term are hidden, unlike traditional schemes, where typically only the exponent is secret. The result is a non-linear cryptographic structure that is inherently more resistant to known attacks. In this configuration, an adversary is presented with two public values, $K_{11}=x^{x}$ and $K_{12}=x^{y}$, and must simultaneously recover the underlying secrets *x* and *y*, which is believed to be infeasible.

No known classical algorithm can efficiently solve such self-referential exponential equations, especially when defined over large finite fields or modulo a large prime. Furthermore, quantum algorithms such as Shor’s algorithm, which are highly effective against classical discrete logarithm problems, do not apply directly in this context, as the equations involve both hidden bases and hidden exponents in a non-linear form. Consequently, recovering the private keys from the public key representation in this scheme constitutes a hard problem for both classical and quantum adversaries.

### 5.5. Secure Signature

Regarding the signature σ (respectively, the subsignature *r*), it can be written as follows:

$$\left\{\begin{array}{c} x^{y}\;\mathrm{mod}\;p=c_{1}\\ x^{z}\;\mathrm{mod}\;p=c_{2} \end{array}\right.$$

where x,y,z are hidden. Based on the OWA assumption, this system of two non-linear equations and three variables can have p−1 possible solutions, and hidden secret keys cannot be retrieved correctly.

In these elements—the sender’s public key $PK_{S}$, subsignature *r*, and signature σ—the attacker cannot separate the base or the exponent in a way that would allow them to uncover any of the secret keys.

**Figure 1:**
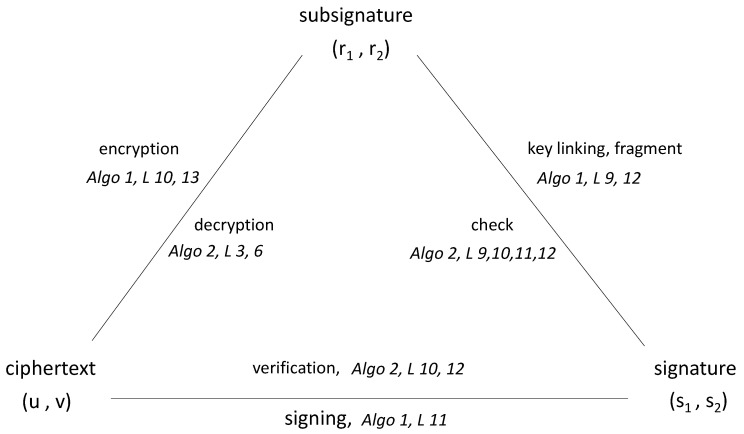
A closed connection circuit to ensure security and prevent signature forgery.

Figure 1 illustrates the internal structure and flow of the proposed PQES scheme, highlighting the tight coupling between encryption, signing, and verification. Calculating the ciphertext (u,v), the signature $\left(s_{1},s_{2}\right)$, and the subsignature $\left(r_{1},r_{2}\right)$ forms a closed cryptographic loop. This loop is non-trivial and can only be consistently completed by a legitimate sender who possesses the correct secret keys $\left(k_{1},k_{2}\right)$. The arrows represent the operational flow defined in Algorithm 1, where key fragments are used to produce components of the signature and subsignature in a mutually dependent manner.

During decryption (Algorithm 2), the receiver uses their secret key to recover the values necessary for message reconstruction and verification. The verification phase performs a series of interlinked algebraic checks involving all components, $\left(s_{1},s_{2}\right)$, $\left(r_{1},r_{2}\right)$, and (u,v), ensuring that the entire structure is cryptographically coherent. This circular dependency ensures message authenticity and integrity and binds the encrypted message to the signer’s identity in a way that is computationally infeasible to forge without access to the signing keys.

The attacker’s challenge resulting from the connection circuit could be summarized as follows:

(5) $$\left\{\begin{array}{c} u=r_{1}\left(a-m\right)\\ v=r_{1}\left(b+r_{2}\right)\\ r_{1}r_{2}=s_{1}^{s_{2}}K_{11}^{h+2s_{1}+r_{1}}\\ s_{1}s_{2}=uK_{11}^{r_{1}+h}\\ s_{1}r_{1}K_{12}^{h}=K_{11}^{s_{1}+h^{\prime }+2h}\\ s_{2}r_{2}=us_{1}^{s_{2}}K_{11}^{s_{1}+2r_{1}-h^{\prime }}K_{12}^{h} \end{array}\right.$$

In (5), where $r_{1},r_{2},u,v$ are related (lines 1 and 2), the attacker has to choose random values for $s_{1}$ and then compute $r_{1},u$ because $r_{1}=f\left(s_{1}\right)$ and $u=f(r_{1})$. Next, he will compute $s_{2}$ and then $r_{2}$ because $s_{2}=f\left(u,r_{1}\right)$ and $r_{2}=f\left(s_{2}\right)$. In the end, the other equations do not hold (Algorithm 2 lines 9, 10, 11, and 12) due to the use of random values for $s_{1}$ and the secret key $k_{1}$ being unknown.

For (5), it does not matter which line the attacker starts from (choosing $r_{1},r_{2}$ verifying Algorithm 2 line 9, choosing $s_{1},s_{2}$ verifying Algorithm 2 line 10, etc.), but he will eventually reach non-identical values, meaning that the last line he reaches will not be satisfied, and this is the meaning of the closed connected circle.

### 5.6. Security Analysis in the Quantum Random Oracle Model (QROM)

We consider two essential properties: confidentiality and authenticity. Confidentiality is addressed through IND-CCA2 security of the encryption component, while authenticity is ensured via SUF-CMA security of the signature component. The proofs are based on two hardness assumptions: a scalar adaptation of the learning-with-errors (LWE) problem and the OWA assumption defined over scalar exponentiations.

**Definition 1** (IND-CCA2 in QROM). *A public-key encryption scheme is IND-CCA2 secure in the QROM if no quantum polynomial-time (QPT) adversary can distinguish the encryption of two chosen messages, even with access to a quantum decryption oracle, except with negligible advantage.*

**Theorem 1.**  *If the scalar-LWE problem is hard for quantum polynomial-time adversaries, then the proposed PQES scheme achieves IND-CCA2 security in the QROM.*

**Proof.**  Let A be a QPT adversary that can break IND-CCA2 security of the scheme with a non-negligible advantage ϵ. To solve an instance of scalar LWE, a QPT reduction B is constructed that uses A.

B receives a scalar-LWE challenge and sets up the public parameters of PQES using this instance. It then simulates the encryption and decryption oracles for A using a quantum-accessible random oracle *H* and controlling query transcripts. During the challenge phase, B embeds the LWE challenge into the ciphertext construction and forwards the challenge ciphertext to A. After receiving the guess $b^{\prime }$, B extracts information from A’s behavior to distinguish whether the ciphertext corresponds to $m_{0}$ or $m_{1}$, thereby solving the scalar-LWE problem with the advantage related to ϵ.

Since the scalar-LWE structure is hidden in the ciphertext and the random oracle is quantum-accessible, the simulation is consistent in the QROM. Hence, the existence of A contradicts the scalar-LWE hardness assumption. □

**Definition 2** (SUF-CMA in QROM). *A signature scheme is strongly unforgeable under chosen message attacks (SUF-CMA) if no QPT adversary can produce a valid signature on a new message or alter a valid signature on a previously signed message, even after querying the signer on messages of its choice, with more than negligible probability.*

**Theorem 2.**  *If the OWA assumption holds in the QROM, then the proposed PQES scheme achieves SUF-CMA security.*

**Proof.**  Suppose a QPT adversary A breaks SUF-CMA with non-negligible probability ϵ. To invert the one-way function underlying the generation of public keys, a QPT reduction B is constructed that uses A, computing the secret keys $\left(k_{1},k_{2}\right)$ from the public values $K_{11}=k_{1}^{k_{1}}$ and $K_{12}=k_{1}^{k_{2}}$.

B simulates the signing oracle for A by programming the quantum random oracle and using knowledge of partial secret values embedded in the challenge. If A produces a new valid signature $\left(s_{1},s_{2}\right)$ on a message $m^{*}$ that was not previously queried (or modifies an existing signature), then due to the strong binding between the signature values and the secret keys, B extracts information that breaks the OWA assumption.

This contradiction implies that the PQES scheme is SUF-CMA secure in the QROM, assuming the hardness of inverting scalar self-exponentiation functions. □

### 5.7. Resistance to Known Attacks

Lattice reduction attacks: PQES does not rely on lattice-based constructions such as LWE over high-dimensional vectors or matrix structures. Instead, it is built over scalar integer arithmetic with non-linear exponentiation operations, specifically involving forms such as $x^{x}$ and $x^{y}\;\mathrm{mod}\;p^{\prime }$. As a result, attacks that exploit the lattice structure, such as basis reduction algorithms such as BKZ or LLL, do not apply to PQES. There is no hidden linear structure or short vector problem to exploit, which protects the scheme from classical lattice reduction attacks.

Quantum search attacks: While the PQES scheme avoids vulnerabilities to Shor’s algorithm due to the absence of discrete log-like group structures, Grover’s algorithm may still apply to brute-force key or preimage search. In particular, Grover’s algorithm can quadratically speed up exhaustive search over *n*-bit secrets, reducing the effective security level from *n* to approximately n/2. To maintain post-quantum security, key sizes and hash functions that offer at least 256-bit classical security are chosen, yielding approximately 128-bit security against quantum search.

Key reuse: The design of PQES incorporates message-dependent randomness and signature components $\left(s_{1},s_{2}\right)$ that vary per message through hash-derived values and random selection of fragments $a_{1}$, $b_{1}$, $a_{2}$, and $b_{2}$ where $k_{i}=a_{i}+b_{i}$. Although the sender’s secret keys $\left(k_{1},k_{2}\right)$ remain fixed, the use of randomized fragmentation and fresh hashing per message prevents replay attacks and leakage. Notably, repeated use of the same public keys does not expose any algebraic structure that would allow key recovery or message correlation due to the non-linearity and hash-driven variation in each signing/encryption session.

## 6. Performance Analysis

The implementation uses SHA-256 as a cryptographic hash function. It is modeled as a quantum-accessible random oracle in the security proofs and offers 128-bit preimage resistance, which is sufficient under NIST Level 1. The use of h=Hash(m) and $h^{\prime }=\mathrm{Hash}\left(h\right)$ is intended to increase entropy and avoid structure in hash inputs. The choice of scalar values such as modulus $p,p^{\prime }$ and secret keys $\left(k_{1},k_{2}\right)$ directly impacts both security and performance. To align with NIST Level 1 quantum security, a 256-bit prime for $p^{\prime }$ is selected. This ensures 128-bit resistance to quantum attacks such as Grover’s search, while allowing efficient modular arithmetic on 32-bit or 64-bit embedded processors.

The security of PQES also depends on fresh randomness per session, particularly for the scalar *x* and the fragmentation of $k_{2}$ into $\left(a_{2},b_{2}\right)$. A quantum-safe cryptographic random number generator is assumed. These random values must not be reused or biased. The design ensures that each encryption/signature session uses unique and independent randomness, and any deviation would be detectable or would fail securely. Given these parameters, the scheme targets NIST Level 1 security. For higher levels (e.g., Level 3 or 5), larger primes and key lengths (e.g., 384 or 512 bits) may be used.

According to the illustration of parameter selection, the sizes of the ciphertext and signature depend on *p*, which is actually dependent on two main factors: the security parameter $p^{\prime }$ (the smallest size in the illustration) and the plaintext *m*; the other parameters can be chosen accordingly.

In choosing $p^{\prime }$ and *m* lengths of 257 bits and 200 bits respectively, a prime number *p* of 995 bits is sufficient. This gives a ciphertext (u,v) of about 2 kbit and a signature ($s_{1},s_{2}$) equal to 512 bits. With $p^{\prime }$ of 257 bits, the lengths of the remaining parameters are as follows: receiver sk (s,e) is (265 bits, 731 bits), receiver pk (a,b) is (995 bits, 995 bits), sender sk ($k_{1},k_{2}$) is 512 bits, and sender pk ($k_{11},k_{12}$) is 512 bits.

Test environment: CPU, Intel Core i7-10610U 2.30 GHz; RAM, 16 GB DDR4; storage, SSD; operating system, Windows 11; and programming language, Python 3.10.

Table 2 presents a comparative evaluation of the execution times required for signcryption and unsigncryption operations across five schemes, including the proposed PQES construction. The measured values provide important information about the practical efficiency of the schemes, particularly in contexts where computational resources are limited or real-time performance is critical.

The signcryption scheme of Ref. [17], although within a symmetric cryptosystem framework, achieves impressively low computational overhead. With signcryption and unsigncryption times of 0.00015 and 0.00027 s, respectively, it outperforms most existing methods in terms of speed. However, the symmetric nature of the design and the lack of post-quantum resistance (as noted in Table 1) limit its applicability in future-proof cryptographic deployments.

The elliptic curve-based signcryption scheme in Ref. [18], although originally designed for efficiency in certificateless environments, exhibits significantly higher execution times: approximately 1.62 s for signcryption and 2.43 s for unsigncryption. The relatively slow performance can be attributed to the reliance on elliptic curve cryptographic operations and the inclusion of hybrid techniques to mitigate key escrow issues. Moreover, as the scheme is not quantum-resistant, its practical relevance in the post-quantum context is diminished despite its classical security model.

The isogeny-based signcryption scheme in Ref. [16] is notable for its strong post-quantum security guarantees. However, this comes at a substantial cost in performance, with both signcryption and unsigncryption operations requiring approximately 2580 s (i.e., over 43 min). Such high computational latency stems from the intrinsic complexity of isogeny-based operations and the multi-stage design leveraging three hard mathematical problems: commutative supersingular isogeny decisional Diffie–Hellman, group action inverse problem, an knowledge of exponent. This renders the scheme impractical for real-time applications or deployment in constrained environments, although it remains of theoretical significance.

CRYSTALS-Kyber and Dilithium are evaluated as a combined signcryption process (encrypt + sign). Values are derived from the NIST reference implementations. Kyber512’s encryption time = 3.3 ms, and Dilithium2’s signature time = 5.6 ms. So, the total signcryption = 8.9 ms. Similarly, unsigncryption = 3.3 + 7.9 ms = 11.2 ms.

PQES, the proposed scheme, achieves an excellent trade-off between performance and security. With a signcryption time of only 0.00077 s and an unsigncryption time of 0.0011 s, the scheme maintains extremely low computational overhead within the same order of magnitude as Ref. [17], yet with the critical advantage of being quantum-resistant. The scalar-integer-based construction, derived from a modified LWE assumption and implemented without the complexity of lattice or isogeny structures, provides a lightweight and efficient cryptographic primitive. These features make PQES particularly suitable for constrained devices such as IoT nodes, mobile systems, and embedded controllers.

**Figure 2:**
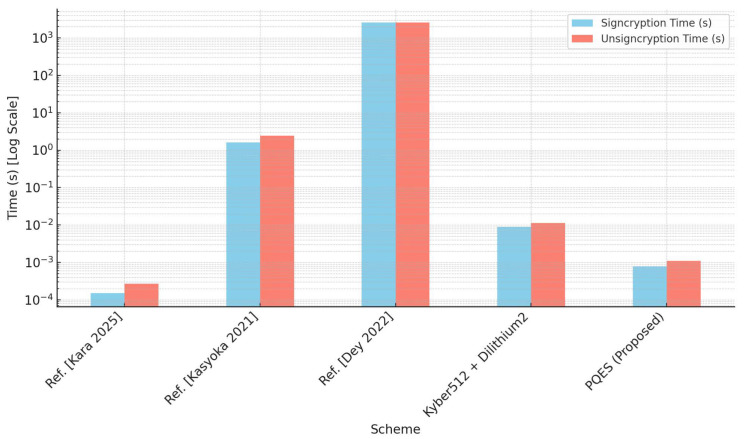
Execution time comparison (log scale) of our method with the techniques [16,17,18], and CRYSTALS-Kyber512 + Dilithium2.

This comparison underscores the primary advantage of the PQES scheme (Figure 2), which offers post-quantum security with a minimal time footprint. Unlike most PQ alternatives that introduce significant overhead, PQES maintains compatibility with lightweight platforms and real-time requirements.

### Trade-Offs and Limitations

The use of scalar integer arithmetic brings significant benefits in terms of simplicity, speed, and memory efficiency, particularly for constrained environments. However, this design choice also introduces certain trade-offs. First, scalar-based constructions do not benefit from the algebraic structure of lattices or codes, which can sometimes enable tighter security reductions or richer functionality (e.g., homomorphic encryption or advanced key sharing). Second, ensuring strong hardness assumptions (such as the OWA assumption) in purely scalar settings may require larger parameters to compensate for the lack of structured noise used in lattice-based schemes. Finally, while the exponentiation-based design is fast in software, it may be less favorable than lattice schemes when vectorized or hardware-accelerated environments are considered. These trade-offs are consciously made to prioritize simplicity and post-quantum feasibility over advanced algebraic features.

While the PQES scheme demonstrates strong efficiency and post-quantum security within the scalar integer framework, some limitations and areas for improvement should be acknowledged. First, the security of the scheme relies on relatively new assumptions, such as the OWA and a scalar version of LWE, which may benefit from further cryptanalytic scrutiny. Second, while scalar arithmetic is efficient for small-to-medium-scale messages, scalability to very large data or streaming contexts may require additional padding, fragmentation, or integration with symmetric encryption layers. Furthermore, the current implementation does not yet support advanced multi-user features such as broadcast/multi-recipient encryption and hierarchical key management or batch processing, which could be essential for deployment in large-scale distributed systems. Finally, formal composability and resistance to side-channel attacks on constrained hardware remain important areas for future study.

## 7. Conclusions

In this work, we introduce a novel post-quantum public-key encryption and signature (PQES) scheme designed to meet the dual demands of quantum resilience and computational efficiency. The scheme is based on a simplified scalar-integer framework, avoiding high-dimensional structures such as lattices, rings, or isogenies. This minimalistic design significantly reduces both computational and memory overhead, making it particularly suitable for resource-constrained environments such as IoT and mobile platforms.

Security is rooted in a scalar-based adaptation of the learning-with-errors (LWE) problem, formulated under the OWA assumption. Moreover, the scheme tightly integrates encryption and signature functionalities into a single operation, ensuring confidentiality, authenticity, and integrity with minimal performance cost.

Performance evaluation confirms the practical viability of PQES. Specifically, its implementation achieves a signcryption time of 0.00077 s and an unsigncryption time of 0.0011 s. These results are significantly faster than are classical elliptic curve-based schemes, showing a speedup of over 2000× in both operations. Compared to an isogeny-based post-quantum signcryption scheme, which requires approximately 2580 s per operation (as cited in [16]), PQES demonstrates a dramatic performance advantage, confirming the effectiveness of scalar-based cryptographic constructions.

As future work, we plan to explore its extension to multi-recipient and broadcast encryption scenarios while preserving efficiency and security.

## Figures and Tables

**Table 1:** Cryptographic Schemes in the literature. QR: quantum-resistant; Limitations: known implementations or theoretical drawbacks.

Scheme	Category	Type	QR	Limitations
CRYSTALS-Kyber	Lattice-based	Encryption	Y	High memory usage; large key sizes; matrix operations
Classic McEliece	Code-based	Encryption	Y	Very large public key; limited efficiency for constrained devices
BIKE	Code-based	Encryption	Y	Vulnerable to decoding attacks; large ciphertexts
HQC	Code-based	Encryption	Y	High bandwidth requirements; slow decryption on low-end devices
CRYSTALS-Dilithium	Lattice-based	Signature	Y	Moderate signature size; high computational load
Falcon	Lattice-based	Signature	Y	Complex implementation; difficult constant-time realization
SPHINCS+	Hash-based	Signature	Y	Large signature size; slow signing speed
Ref. [17]	Discrete log (scalar integer)	Signcryption	N	Not quantum-resistant; relies on classical DLP
Ref. [18]	Elliptic curve (scalar integer)	Signcryption	N	Vulnerable to Shor’s algorithm; not QR
Ref. [16]	Isogeny	Signcryption	Y	Computationally expensive; security assumptions still evolving

**Table 2:** Execution Time comparison of signcryption and unsigncryption operations. Times are averaged over 100 runs.

Scheme	Signcryption Time (s)	Unsigncryption Time (s)
Ref. [17]	0.00015	0.00027
Ref. [18]	1.62	2.43
Ref. [16]	2580	2580
CRYSTALS-Kyber512 + Dilithium2	0.0089	0.0112
PQES (Proposed)	0.00077	0.0011

## Data Availability

Data are contained within the article.
